# Retraction Note: Exosomes from the tumour-adipocyte interplay stimulate beige/brown differentiation and reprogram metabolism in stromal adipocytes to promote tumour progression

**DOI:** 10.1186/s13046-023-02594-4

**Published:** 2023-01-12

**Authors:** Qi Wu, Juanjuan Li, Zhiyu Li, Si Sun, Shan Zhu, Lijun Wang, Juan Wu, Jingping Yuan, Yimin Zhang, Shengrong Sun, Changhua Wang

**Affiliations:** 1 Department of Breast and Thyroid Surgery, Renmin Hospital of Wuhan University, 238 Ziyang Road, Wuhan, 430060 Hubei Province People’s Republic of China; 2 Department of Clinical Laboratory, Renmin Hospital of Wuhan University, Wuhan, Hubei People’s Republic of China; 3 Department of Pathology, Renmin Hospital of Wuhan University, Wuhan, Hubei People’s Republic of China; 4 Department of Pathophysiology, Wuhan University School of Basic Medical Sciences, Wuhan, 430060 Hubei Province People’s Republic of China

**Retraction Note:** ***J Exp Clin Cancer Res*** **38, 223 (2019)**

**https://doi.org/10.1186/s13046-019-1210-3**

The Editor-in-Chief has retracted this article. After publication concerns were raised regarding irregularities present in multiple figures in this article. There is overlap in Fig. 2A with Fig. 2A of a previously published article [1]. Figure 5A also overlaps with Fig. 3A from a previously published article [1]. Figure 2A overlaps with an image present in Fig. 6C. The Editor-in-Chief therefore, no longer has confidence in the reliability of the results presented in this article. All authors agree to this retraction.
